# Integrative Analysis of Sirtuins and Their Prognostic Significance in Clear Cell Renal Cell Carcinoma

**DOI:** 10.3389/fonc.2020.00218

**Published:** 2020-02-25

**Authors:** Ying Tan, Bijuan Li, Fang Peng, Guanghui Gong, Ning Li

**Affiliations:** ^1^Department of Blood Transfusion, Xiangya Hospital, Central South University, Changsha, China; ^2^NHC Key Laboratory of Cancer Proteomics, Xiangya Hospital, Central South University, Changsha, China; ^3^Department of Pathology, Xiangya Hospital, Central South University, Changsha, China

**Keywords:** sirtuins, epigenetics, TCGA, cancer, clear cell renal cell carcinoma

## Abstract

Sirtuins, class III histone deacetylases, are involved in multiple biological processes in cancer initiation and progression. However, the diverse expression patterns and prognostic values of sirtuins in cancers have yet to be elucidated. In this study, we first evaluated the expression and prognostic values of sirtuins in multiple cancer cohorts using publicly available TCGA pan-cancer datasets. Pan-cancer survival analysis indicated that 6 out of 7 sirtuin family members were significant associated with prognosis of clear cell renal cell carcinoma (KIRC) patients. SIRT1, SIRT3, SIRT4, and SIRT5 were associated with favorable prognosis of KIRC patients, while SIRT6 and SIRT7 were associated with unfavorable prognosis. The expression levels of SIRT4 and SIRT5 in KIRC tissues were lower than that in normal tissues, while SIRT6 and SIRT7 were higher in KIRC tissues. The expression levels of SIRT1, SIRT3, SIRT5, SIRT6, and SIRT7 were significantly correlated with tumor stage and histological grade. DNA methylation may contribute to the dysregulation of sirtuins. Finally, GSEA was conducted to predict the potential functions of sirtuins in KIRC. Our results may provide novel insights for the development of sirtuins-based cancer therapy in KIRC.

## Introduction

Renal cell carcinoma (RCC) is the seventh most common cancer in the developed world, accounting for 2–3% of the total human cancer burden in 2012 (1). RCC can be classified into several histopathological entities, of which clear cell renal cell carcinoma (KIRC) is the most frequent subtype of RCC (75–80%) (2). Surgical resection is an effective method for the treatment of RCC. However, the prognosis of RCC remains unsatisfactory due to late diagnosis and recurrence. Therefore, it is critical to find predictive biomarkers for early diagnosis and prognosis. Epigenetic alterations are an essential event in the development and progression of RCC. They are considered as potential biomarkers for the early diagnosis of RCC and prediction of prognosis (3).

Sirtuins are NAD^+^ dependent deacetylases that belong to the class III histone deacetylase family. Mammalian sirtuins consist of seven members (SIRT1-SIRT7), which all have a conserved core NAD^+^ binding domain, but have different N-terminal and C-terminal domains. The seven members have different catalytic activity, target proteins, and functions (4). Sirtuins are involved in the development and progression of aging and various aging-related disease, including cardiovascular disease, diabetes, and neurodegeneration (5). Kidney disease is part of the main aging-related diseases. Aberrant expressions and functions of sirtuins have been reported in acute and chronic kidney disease. SIRT1 exerts renal protective effects through inhibiting fibrosis, cell apoptosis and inflammation, and regulation of blood pressure. SIRT3 attenuates mitochondrial dysfunction and ameliorates oxidative stress to protect against acute kidney injury (6). Recently, a growing number of studies have found that sirtuins are involved in various biological processes related to tumorigenesis, such as cancer-associated metabolic pathway alterations, uncontrolled proliferation, genome instability, tumor microenvironment. Sirtuins are supposed to have complex roles in human cancers, and they act as both oncogene and tumor-suppressor depending on cancer types and experimental conditions (7). Several studies have been conducted to explore the clinical significance of sirtuins in RCC, however, the results are inconsistent. The protein expression of SIRT1, SIRT3, and SIRT6 have been reported to be significantly lower in human RCC tissues compared with that in adjacent normal tissues, and high protein expression levels of SIRT1 and SIRT3 were found to be significantly associated with better survival in RCC patients (8, 9). However, SIRT1 was also reported to be up-regulated in RCC and the expression of SIRT1 was significantly associated with poor prognosis in RCC patients (10). Knockdown of SIRT1 suppressed cell proliferation and promoted apoptosis in RCC cell lines (11). SIRT2 mRNA and protein expression were significantly elevated in RCC tissues compared with normal tissues, and high SIRT2 expression is associated with more advanced tumors and poor prognosis (12). SIRT3 overexpression inhibited the growth of RCC cell lines and enhanced mitochondrial biogenesis (13). However, SIRT3 was reported to be up-regulated in the mitochondrial fraction of human RCC tissues, and knockdown of SIRT3 inhibited RCC cell proliferation and tumor growth *in vivo* (14). SIRT5 promotes KIRC tumorigenesis through inhibiting SDHA succinylation and silencing SIRT5 inhibited RCC cell proliferation (15).

To date, the expression pattern, prognostic significance, and biological function of sirtuins in KIRC have not yet been fully elucidated. In this study, we extended the knowledge on KIRC by using the gene expression data and clinic data from the public database online. We conducted a comprehensive analysis of the relationship between the expression of sirtuins and clinicopathological parameters of KIRC patients, and conducted a preliminary analysis of their regulation and potential functions.

## Methods

### TCGA Datasets

The RNA-seq data and clinical information were downloaded from TCGA (https://portal.gdc.cancer.gov/). The 16 cancer types selected contained at least 9 samples in the normal group. The genomic alteration of sirtuins was assessed using the cBioPortal (16, 17). Analysis of DNA methylation between normal tissues and KIRC cancer tissues was performed using UALCAN (18).

### GSEA

GSEA was carried out to identify the potential underlying mechanisms of sirtuins expression on the pathogenesis and prognosis of KIRC (19, 20). The gene sets were obtained from the MSigDB database v6.2.Gene set. Permutations were performed 1,000 times for each analysis. The nominal *p*-value (NOM *p* < 0.05) and False discovery rate (FDR *q* < 0.25) were used to select significantly enriched gene sets.

### Statistical Analysis

**Figure 1:**
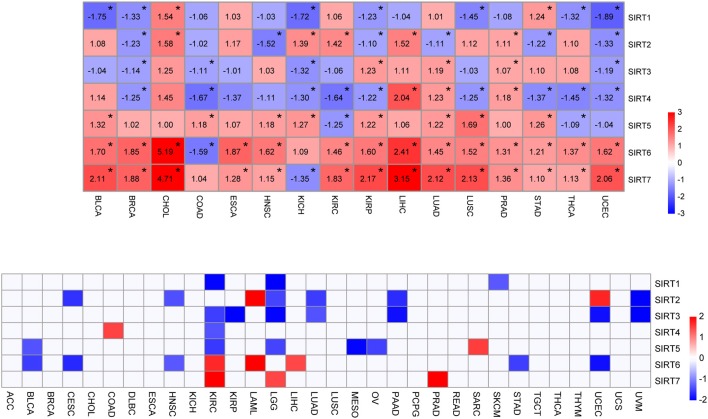
Expression level and survival analysis of sirtuins across TCGA cancer types. **(A)** Heatmap of sirtuins with changed expressions in 16 cancer cohorts compared to normal tissues (*P < 0.05). **(B)** Summary of hazard ratios (HR) illustrating cancer-sirtuin pairs with altered prognosis. ACC, Adrenocortical Carcinoma; BLCA, Bladder Urothelial Carcinoma; BRCA, Breast Invasive Carcinoma; CESC, Cervical Squamous Cell Carcinoma and Endocervical Adenocarcinoma; CHOL, Cholangiocarcinoma; COAD, Colon Adenocarcinoma; DLBC, Lymphoid Neoplasm Diffuse Large B-cell Lymphoma; ESCA, Esophageal Carcinoma; HNSC, Head and Neck Squamous Cell Carcinoma; KICH, Kidney Chromophobe; KIRC, Kidney Renal Clear Cell Carcinoma; KIRP, Kidney Renal Papillary Cell Carcinoma; LAML, Acute Myeloid Leukemia; LGG, Brain Lower Grade Glioma; LIHC, Liver Hepatocellular Carcinoma; LUAD, Lung Adenocarcinoma; LUSC, Lung Squamous Cell Carcinoma; MESO, Mesothelioma; OV, Ovarian Serous Cystadenocarcinoma; PAAD, Pancreatic Adenocarcinoma; PCPG, Pheochromocytoma and Paraganglioma; PRAD, Prostate Adenocarcinoma; READ, Rectum Adenocarcinoma; SARC, Sarcoma; SKCM, Skin Cutaneous Melanoma; STAD, Stomach Adenocarcinoma; TGCT, Testicular Germ Cell Tumors; THCA, Thyroid carcinoma; THYM, Thymoma; UCEC, Uterine Corpus Endometrial Carcinoma; UCS, Uterine Carcinosarcoma; UVM, Uveal Melanoma.

**Figure 2:**
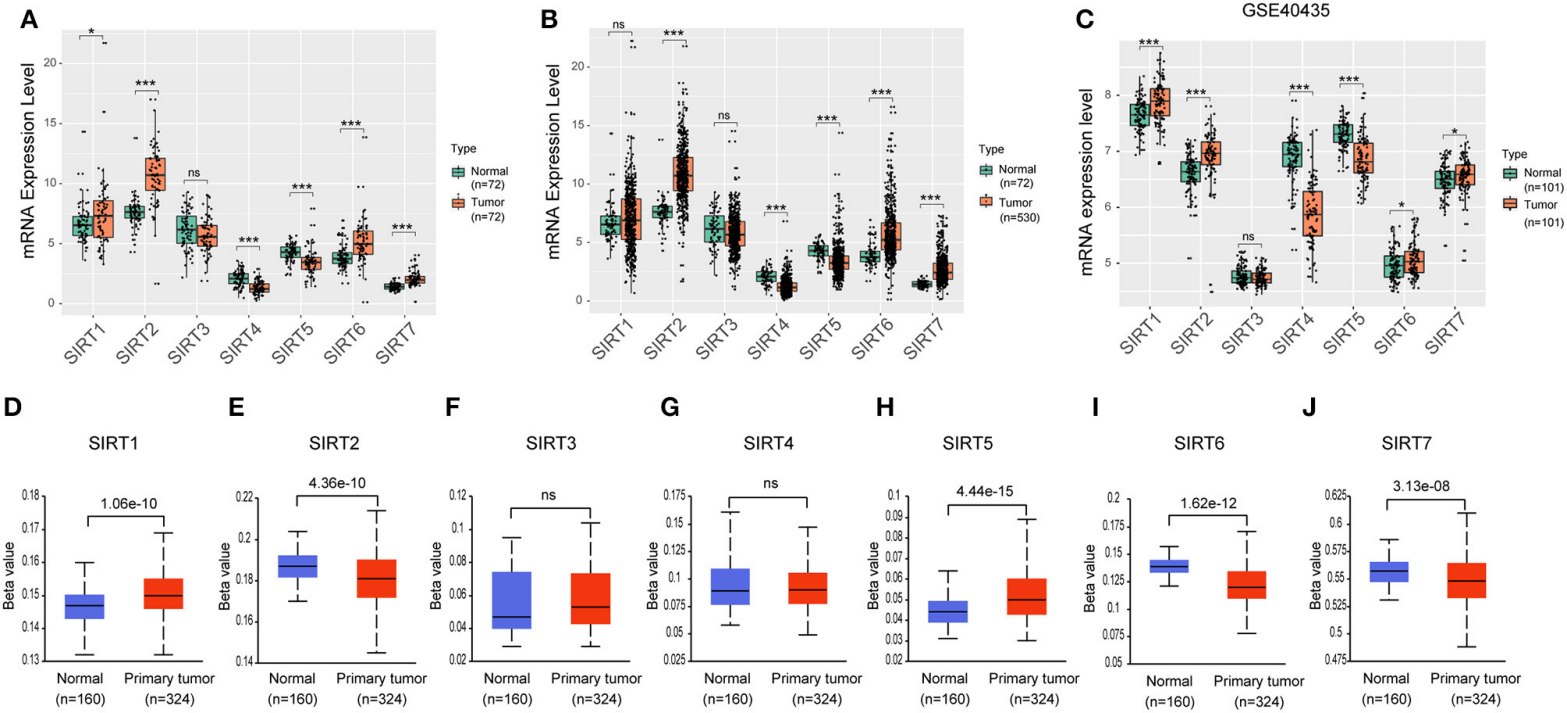
Expression level of sirtuins in KIRC. Boxplots show sirtuins expression in normal tissues compared with paired cancer tissues **(A)**, or unpaired KIRC tissues **(B)**. **(C)** Boxplots show sirtuins expression in normal and KIRC tissues from GSE40435. **(D–J)** Promoter methylation status of sirtuins in KIRC (*P < 0.05, ***P < 0.001).

**Figure 3:**
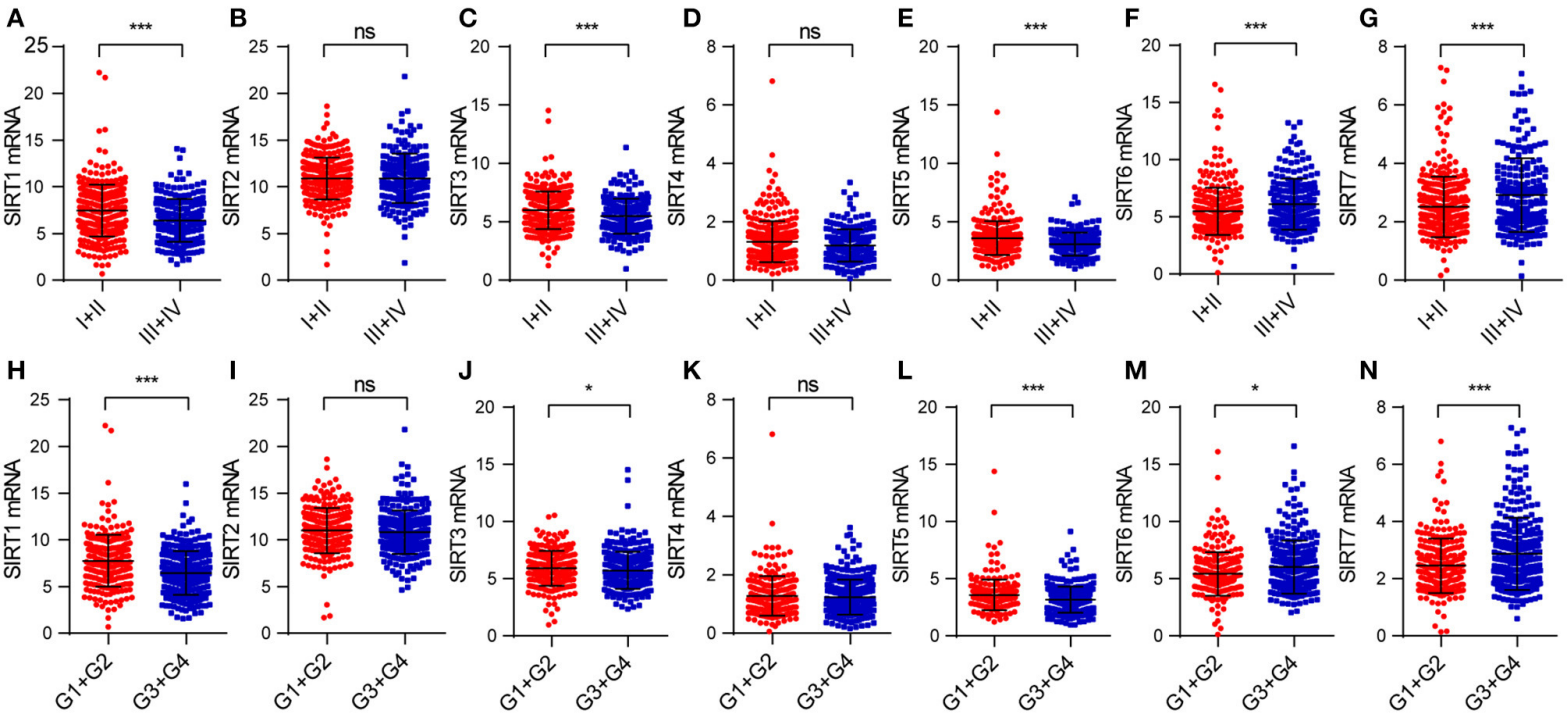
The relationship between sirtuins expression and clinicopathological features in KIRC patients: **(A–G)** pathological stage, **(H–N)** histological grade (*P < 0.05, ***P < 0.001).

All data analysis was conducted using SPSS Version 20 software or Graphpad Prism 7.0. In **Figures 2A,C**, we compared the expression of each sirtuin between tumor tissues and corresponding paired normal tissues, using the paired *t*-test or Wilcoxon matched-pairs signed test. In Figures 1A, 2B, 3, when two groups were with normal distribution, we used the standard Student's test for equal variance or Welch *t*-test for unequal variance. Otherwise, we used the Mann-Whitney *U*-test (non-normal distribution). In Table 1, patients were divided into low and high groups based on the median value of sirtuins expression. The chi-square and Fisher exact tests were applied to identify the correlations between the sirtuins expression and clinical features of KIRC patients. The Kaplan-Meier curve was used to compare the influence of sirtuins expression on overall survival and disease-free survival, and a log-rank test was used to estimate the difference in survival. A value of *P* < 0.05 was considered statistically significant.

**Table 1:** The Correlation between sirtuins and clinicopathological parameters in KIRC.

**Parameters**	***N***	**SIRT1**			**SIRT2**			**SIRT3**			**SIRT4**			**SIRT5**			**SIRT6**			**SIRT7**		
		**Low**	**High**	***p***	**Low**	**High**	***p***	**Low**	**High**	***p***	**Low**	**High**	***p***	**Low**	**High**	***p***	**Low**	**High**	***p***	**Low**	**High**	***p***
**GENDER**																						
Female	186	90 (48.4%)	96 (51.6%)	0.585	89 (47.8%)	97 (52.2%)	0.467	74 (39.8%)	112 (60.2%)	0.001	101 (54.3%)	85 (45.7%)	0.145	77 (41.4%)	109 (58.6%)	0.004	94 (50.5%)	92 (49.5%)	0.856	86 (46.2%)	100 (53.8%)	0.203
Male	344	175 (50.9%)	169 (49.1%)		176 (51.2%)	768 (48.8%)		191 (55.5%)	153 (44.5%)		164 (47.7%)	180 (52.3%)		188 (54.7%)	156 (45.3%)		171 (49.7%)	173 (50.3%)		179 (52.0%)	165 (48.0%)	
**AGE**																						
≤60 years	264	126 (47.7%)	138 (52.3%)	0.297	131 (49.6%)	133 (50.4%)	0.862	137 (51.9%)	127 (48.1%)	0.385	129 (48.9%)	135 (51.1%)	0.602	127 (48.1%)	137 (51.9%)	0.385	127 (48.1%)	137 (51.9%)	0.385	131 (49.6%)	133 (50.4%)	0.862
>60 years	266	139 (52.3%)	127 (47.7%)		134 (50.4%)	132 (49.6%)		128 (48.1%)	138 (51.9%)		136 (51.1%)	130 (48.9%)		138 (51.9%)	128 (48.1%)		138 (51.9%)	128 (48.1%)		134 (50.4%)	132 (49.6%)	
**TNM STAGE**																						
I	265	102 (38.5%)	163 (61.5%)	0.000	130 (49.1%)	135 (50.9%)	0.854	116 (43.8%)	149 (56.2%)	0.041	122 (46.0%)	143 (54.0%)	0.461	112 (42.3%)	153 (57.7%)	0.005	147 (55.5%)	118 (44.5%)	0.027	146 (55.1%)	119 (44.9%)	0.142
II	57	30 (52.6%)	27 (47.4%)		32 (56.1%)	25 (43.9%)		28 (49.1%)	29 (50.9%)		31 (54.4%)	26 (45.6%)		28 (49.1%)	29 (50.9%)		29 (50.9%)	28 (49.1%)		28 (49.1%)	29 (50.95)	
III	123	76 (61.8%)	47 (38.2%)		61 (49.6%)	62 (50.4%)		73 (59.3%)	50 (40.7%)		67 (54.5%)	56 (45.5%)		75 (61.0%)	48 (39.0%)		58 (47.2%)	65 (52.8%)		57 (46.3%)	66 (53.7%)	
IV	82	54 (65.9%)	28 (34.1%)		40 (48.8%)	42 (51.25)		46 (56.1%)	36 (43.9%)		43 (52.4%)	39 (47.6%)		48 (58.5%)	34 (41.5%)		29 (35.4%)	53 (64.6%)		33 (40.2%)	49 (59.8%)	
Unknown	3	3 (100.0%)	0 (0.0%)		2 (66.7%)	1 (33.3%)		2 (66.7%)	1 (33.3%)		2 (66.7%)	1 (33.3%)		2 (66.7%)	1 (33.3%)		2 (66.7%)	1 (33.3%)		1 (33.35)	2 (66.7%)	
**G STAGE**																						
G1	14	3 (21.4%)	11 (78.6%)	0.000	6 (42.9%)	8 (57.1%)	0.500	4 (28.6%)	10 (71.4%)	0.001	6 (42.9%)	8 (57.1%)	0.078	5 (35.7%)	9 (64.3%)	0.000	5 (35.7%)	9 (64.3%)	0.035	5 (35.7%)	9 (64.3%)	0.016
G2	227	85 (37.4%)	142 (62.6%)		113 (49.8%)	114 (50.2%)		105 (46.3%)	122 (53.7%)		109 (48.0%)	118 (52.0%)		97 (42.7%)	130 (57.3%)		125 (55.1%)	102 (44.9%)		130 (57.3%)	97 (42.7%)	
G3	206	116 (56.3%)	90 (43.7%)		99 (48.1%)	107 (51.9%)		97 (47.1%)	109 (52.9%)		97 (47.1%)	109 (52.9%)		108 (52.3%)	98 (47.6%)		101 (49.0%)	105 (51.0%)		93 (45.1%)	113 (54.9%)	
G4	75	56 (74.7%)	19 (25.3%)		44 (58.7%)	31 (41.3%)		53 (70.7%)	22 (29.3%)		49 (65.3%)	26 (34.7%)		54 (72.0%)	21 (28%)		28 (37.3%)	47 (62.7%)		31 (41.3%)	44 (58.7%)	
Unknown	8	5 (62.5%)	3 (37.5%)		3 (37.5%)	5 (62.5%)		6 (75.0%)	2 (25.0%)		4 (50.0%)	4 (50.0%)		1 (12.5%)	7 (87.5%)		6 (75.0%)	2 (25.0%)		6 (75.0%)	2 (25.0%)	

## Results

### Expression Pattern and Survival Analysis of Sirtuins in Pan-Cancer Perspective

To determine the expression pattern and prognostic value of sirtuins across cancer types, we downloaded the clinical information of 32 cancer cohorts and corresponding cancer tissue RNA-seq datasets from the TCGA database. We removed cancer types with <9 samples in the normal tissue group to ensure that there were sufficient samples in each group for statistical analysis. We analyzed the expression of sirtuins in 16 cancer types, including BLCA, BRCA, CHOL, COAD, ESCA, HNSC, KICH, KIRC, KIRP, LIHC, LUAD, LUSC, PRAD, STAD, THCA, and UCEC (Figure 1A). Of all the 112 comparisons between cancers and control tissues, 79 were statistically significant (70.5%). SIRT5, SIRT6, and SIRT7 exhibited up-regulation in multiple cancer types. SIRT6 and SIRT7 were up-regulated in 14 of the 15 significantly altered cancer types. SIRT1 and SIRT4 significantly decreased in 7 cancer types and 9 cancer types, respectively. According to the median value, sirtuins were divided into high-expression and low-expression groups. Kaplan-Meier curve and log-rank test analysis were used for overall survival analysis. An overview of these results was shown in Figure 1B and Supplementary Table 1, there was an apparent heterogeneity between different cancer types. We observed a unique role of sirtuins in KIRC comparing to other cancer types. Six members of sirtuins were significantly associated with overall survival in patients with KIRC, the subsequent studies will focus on the role of sirtuins in KIRC.

### The Expression Level and Promoter Methylation Status of Sirtuins in KIRC

We further investigated the expression of sirtuins in KIRC in more detail. In 72 paired KIRC tissues and corresponding adjacent normal tissues, expressions of SIRT4 and SIRT5 were down-regulated in cancer tissues than in normal tissues, while expressions of SIRT6 and SIRT7 were up-regulated in cancer tissues (Figure 2A). Similar results were shown between 530 KIRC tissues and 72 normal tissues (Figure 2B). We further confirmed these results using an independent KIRC cohort from the GEO database (GSE40435). The expression of SIRT4 and SIRT5 were down-regulated in cancer tissues, while SIRT6 and SIRT7 were up-regulated (Figure 2C). This was consistent with our result that SIRT4 and SIRT5 were favorable prognostic factors for KIRC patients, SIRT6 and SIRT7 were unfavorable. It is clear that promoter methylation plays an important role in renal tumorigenesis by silencing tumor suppressor genes (21). There was a relatively high frequency of promoter methylation as compared to the number of somatic alterations in RCC (22). To determine the methylation status of sirtuins in KIRC tissues, we applied UALCAN to explore the level of methylation in the promoter region and its relationship with sirtuins mRNA expression. The promoter of SIRT1 and SIRT5 were hypermethylated in KIRC tissues than that in normal tissues, while SIRT2, SIRT6, and SIRT7 were hypomethylated in KIRC tissues (Figures 2D–J).

### Correlations of Sirtuins With Clinicopathological Characteristics in KIRC

We explored the correlation between sirtuins expression and clinicopathological parameters of patients with KIRC. In total, we identified 530 KIRC samples with mRNA expression data and clinical information. We applied the chi-square test to identify the correlation between the sirtuins expression and clinicopathological parameters of KIRC patients. SIRT3 and SIRT5 were highly expressed in female patients. Low expressions of SIRT1, SIRT3, and SIRT5 were significantly correlated with advanced TNM stage and poor histological grade. High SIRT6 expression was associated with advanced TNM stage and poor histological grade stage. High SIRT7 expression was associated with poor histological grade (Table 1). We further analyzed the relationship between the expression of sirtuins and tumor stage, histological grade. The result showed that SIRT1, SIRT3, and SIRT5 were lower in advanced TNM stage and poor histological grade, while SIRT6 and SIRT7 were higher (Figure 3). This was consistent with that SIRT1, SIRT3, and SIRT5 might be a favorable factor for KIRC patients, whereas SIRT6 and SIRT7 might be unfavorable.

### Prognostic Value of Sirtuins in KIRC

**Figure 4:**
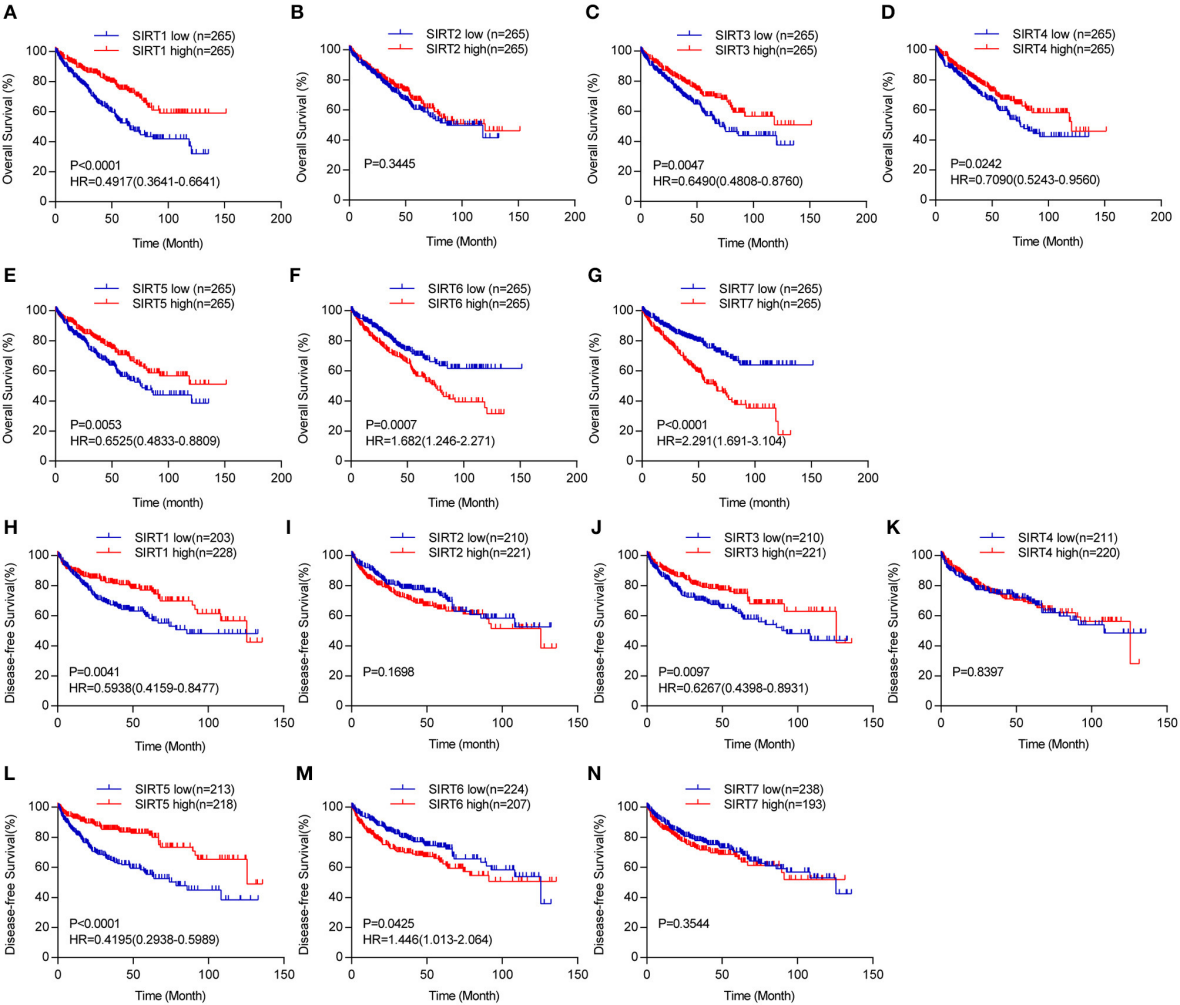
The prognostic value of sirtuins in KIRC. Kaplan-Meier curves show the correlation between sirtuins expression and overall survival **(A–G)**, and disease-free survival **(H–N)** of KIRC patients.

We further explored the prognostic value of sirtuins in patients with KIRC. We assessed the relationship between individual sirtuin and the survival of KIRC patients. Decreased SIRT1, SIRT3, SIRT4, and SIRT5 expressions and increased SIRT6 and SIRT7 expressions were strongly associated with poor overall survival (Figures 4A–G). Further, Kaplan-Meier curves for disease-free survival showed that low SIRT1, SIRT3 and SIRT5, and high SIRT6 were significantly correlated with poor disease-free survival (Figures 4H–N).

### Genomic Alteration of Sirtuins in KIRC

**Figure 5:**
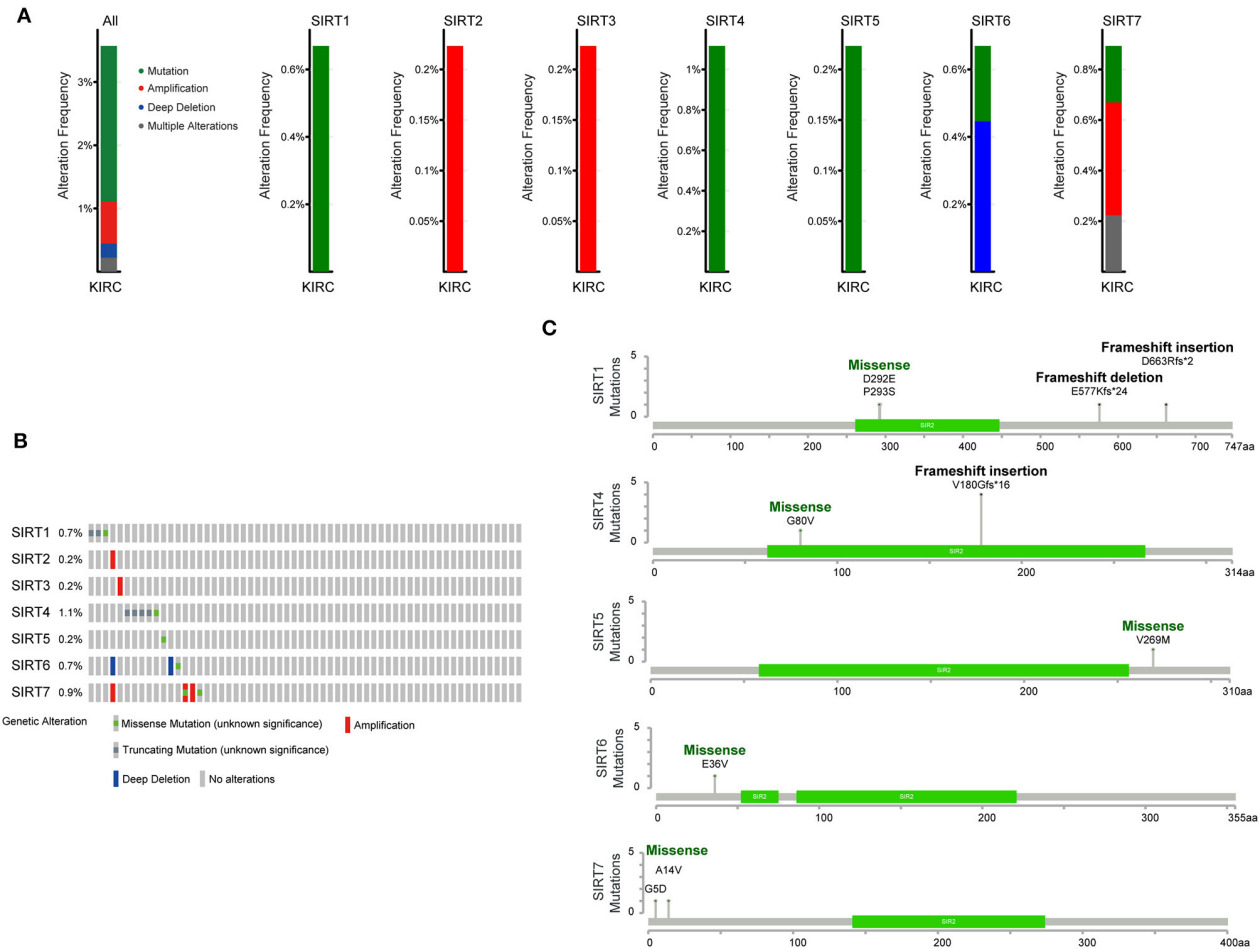
Genomic analysis of sirtuins in KIRC. **(A)** Summary of genomic alteration of sirtuins in KIRC. **(B)** Oncoprint visual summary of alteration on a query of sirtuins. **(C)** The mutations of SIRT1, SIRT4, SIRT5, SIRT6, and SIRT7 were plotted. The SIR2 domain is displayed in green.

We next investigated the potential mechanisms that dysregulate sirtuins expression in KIRC. We applied the cBioPortal online tool for KIRC (The Cancer Genome Atlas, Provisional) to analyze the genetic alteration frequency of sirtuins. A total of 448 patients were analyzed. The oncoprints include missense mutation, deletion, and amplification. The percentage of genetic alterations of sirtuins in KIRC varied from 0.2 to 1.1% (Figures 5A,B). The percentages of genetic mutation in SIRT1, SIRT2, SIRT3, SIRT4, SIRT5, SIRT6, and SIRT7 were 0.67% (mutation), 0.22% (amplification), 0.22% (amplification), 1.12% (mutation), 0.22% (mutation), 0.67% (0.22% mutation, 0.45% deep deletion), 0.89% (0.22% mutation, 0.45% amplification, 0.22 multiple alterations). Missense mutation was identified in SIRT1, SIRT4, SIRT5, SIRT6, and SIRT7, and the mutation in the SIR2 domain was verified in SIRT1 and SIRT4. Besides, frameshift insertion was found in SIRT1 and SIRT4 (Figure 5C).

### Predicted Functions of Sirtuins in KIRC

GSEA was performed to investigate the potential mechanisms of sirtuins in KIRC. Samples were divided into high group and low group according to the median value.

**Figure 6:**
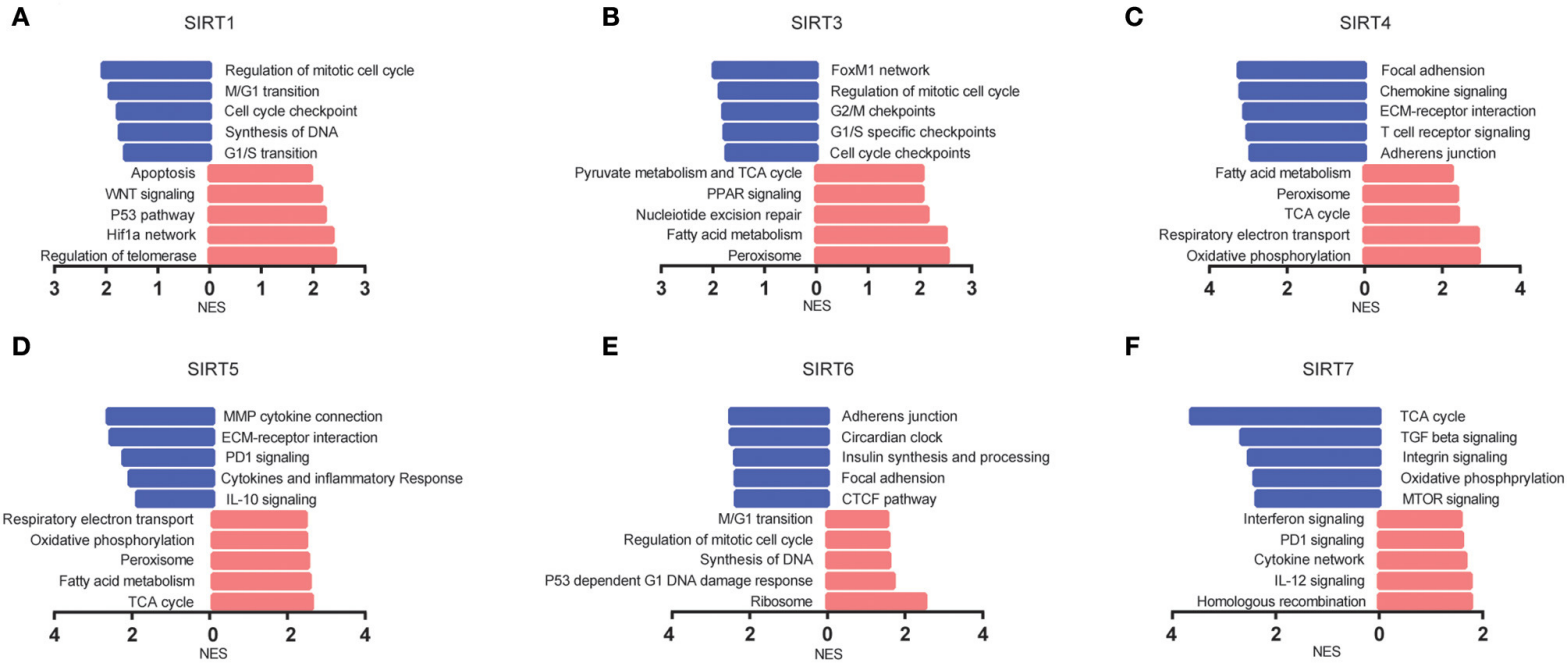
The pathways associated with sirtuins alterations were predicted by GSEA. **(A–F)** Pathway alterations in the high-expression group vs. low-expression group. Pathways enriched in phenotype high were shown in red (right), and pathways enriched in phenotype low were shown in blue (left). All gene sets were significantly enriched at nominal *p* < 5% and FDR < 25%.

We analyzed KEGG gene sets using the GSEA. Pathways involving the TCA cycle and oxidative phosphorylation were significantly enriched and positively correlated with SIRT3, SIRT4, and SIRT5, whereas negatively correlated with SIRT7. The well-known pathways associated with cell cycle were significantly regulated by sirtuins alteration in KIRC (Figure 6).

## Discussion

Sirtuins are involved in a few cancer-related biological processes, including cancer metabolism, genome stability, and tumor microenvironment. Sirtuins act as tumor suppressors or tumor promoters depending on the cellular context and experimental conditions. Due to the complex role of sirtuins in cancer, a better understanding of each sirtuin will be important for the future development of sirtuins-based therapy. Our results showed that sirtuins are heterogeneous in different cancers. According to the pan-cancer survival analysis and expression profiling analysis of TCGA, 6 out of 7 sirtuin family members were significantly correlated with the survival of KIRC patients. Next, we found that the expression of sirtuins was related to the histological grade and pathological stage of KIRC. We found that promoter methylation status may contribute to the dysregulation of sirtuins in KIRC.

SIRT1, the best known and most studied member of the sirtuins family, was reported to play a dual role in tumorigenesis. SIRT1 plays a tumor-suppressor role in various human cancers, including breast cancer, bladder cancer, and glioblastoma (23, 24). Meanwhile, overexpression of SIRT1 was also reported in several cancers and associated with an unfavorable prognosis (25, 26). The expression of SIRT1 was reported to predict shorter overall survival, disease-free survival, and cancer-specific survival in KIRC (10). SIRT1 act as a direct target of miRNA-200a and miRNA-22, miRNA-200a and miR-22 could inhibit cell proliferation, arrested cell cycle progression, and promoted cell apoptosis in RCC cell lines (11, 27). However, it was also reported that SIRT1 expression was significantly lower in RCC than in normal tissues and high expression of SIRT1 was correlated with a better prognosis for RCC patients (8). SIRT1 overexpression inhibited RCC cell line proliferation by repressing FGB expression (9). In our study, the mRNA expression of SIRT1 was up-regulated in KIRC than in normal tissues. Low SIRT1 was associated with high TNM stage and advanced histologic grade. Besides, Kaplan-Meier curves showed that low expression of SIRT1 was significantly correlated with poor overall survival and disease-free survival.

SIRT2 is mainly located in the cytoplasm. Recent studies found that SIRT2 may mainly act tumor suppressor by maintaining genome stability (28, 29). The previous study reported that the higher mRNA and protein expression levels of SIRT2 were found in RCC samples than normal tissues. High SIRT2 levels predicted poor survival of RCC patients (12). In our study, we found that the mRNA level of SIRT2 was higher in KIRC tissues than control tissues. However, SIRT2 did not show a significant association with advanced tumor characters or survival.

SIRT3, SIRT4, and SIRT5 are known as mitochondrial sirtuins. They orchestrate numerous aspects of mitochondrial biology, including redox balance, metabolism homeostasis, and mitochondrial dynamics (30). In agreement with SIRT3 functioning as a tumor suppressor, SIRT3 was a favorable prognostic indicator for multiple cancer in our study, including KIRC, KIRP, LGG, LUAD, PAAD, UCEC, and UVM. SIRT3 was deleted or down-regulated in many types of human cancers (31). In renal cancer, SIRT3 was reported to be down-regulated in KIRC tissues and predicted a favorable survival of KIRC patients (8, 13, 32). Overexpression of SIRT3 in RCC cell lines inhibited cell proliferation and reversed the Warburg effect (13). In our study, although SIRT3 did not show a significant difference between KIRC and normal tissues, SIRT3 expression was inversely correlated with high TNM stage and advanced histological grade in KIRC patients. In addition, low SIRT3 expression was significantly associated with poor overall survival and disease-free survival of KIRC patients, which is consistent with the previous view that SIRT3 act as a tumor suppressor. SIRT4 exerts tumor suppressor activity in many cancers (33). Until now, little was known about the role of SIRT4 in RCC. In this study, SIRT4 expression was lower in KIRC tissues than in normal tissues. SIRT4 expression was significantly and positively correlated with overall survival of KIRC patients. Currently, the role of SIRT5 in cancer has not been widely reported. SIRT5 was overexpressed in non-small cell lung cancer and colorectal cancer, high SIRT5 expression was an unfavorable predictor of survival. Overexpression of SIRT5 promoted cancer cell growth and drug resistance (34, 35). SIRT5 was recently reported to be down-regulated in glioblastoma and its down-regulation was associated with poor prognosis (35, 36). SIRT5 was reported to be up-regulated in KIRC, SIRT5 promote RCC tumorigenesis by inhibiting SDHA succinylation. In our study, SIRT5 expression decreased in KIRC tissues, and its expression level was negatively correlated with the TNM stage and histological grade in KIRC patients. Lower SIRT5 expression was significantly correlated with poor overall survival and disease-free survival.

SIRT6 has been reported to regulate inflammation, genome instability, glucose metabolism which are associated with tumor suppressor (37). SIRT6 act as a tumor suppressor in several cancers (38), including pancreatic cancer (39), breast cancer (40), and hepatocellular carcinoma (41, 42). While in other cancer types, such as lung cancer (43, 44) and melanoma (45), SIRT6 was up-regulated and act as a tumor promoter. In this study. SIRT6 expression was found to be higher in KIRC tissues than in normal tissues and was significantly and positively correlated with tumor stage and histologic grade. Furthermore, an elevated level of SIRT6 was significantly associated with worse overall survival and disease-free survival in patients with KIRC. Accumulative studies showed that SIRT7 was a biomarker for poor prognosis (46). In agreement with a tumor-promoting role for SIRT7, our study found that SIRT7 was significantly up-regulated in multiple cancers. SIRT7 may be an unfavorable prognostic factor for KIRC and high SIRT7 expression was positively correlated with tumor pathologic stage and histological grade.

It was reported that profound changes in cellular metabolism take place during KIRC tumorigenesis. Because of the frequent alterations of signaling networks that regulate metabolic behavior, KIRC was described as a “metabolic disease” (47). Cellular metabolism in KIRC involved impaired Krebs cycle and oxidative phosphorylation, and a subsequent Warburg metabolic shift to aerobic glycolysis, and all of those features were associated with poor prognosis (48, 49). However, the molecular mechanisms of these metabolic changes are not fully elucidated. In this study, GSEA analyses showed that sirtuins were closely related to metabolic pathways, such as oxidative phosphorylation, Krebs cycle, fatty acid metabolism. Therefore, the analysis above suggested the functions of sirtuins in cellular metabolism, providing insight into the promising therapeutic target in KIRC. Gender has been considered as an independent risk factor for survival in KIRC patients, with a benefit for women (50). The mechanism of this difference is unclear. According to our results, SIRT3 and SIRT5 were highly expressed in female patients. Further experiments may be helpful to understand the relationship between SIRT3/SIRT5 and gender in KIRC. In general, our study investigated the expression pattern and prognosis role of sirtuins in KIRC by using TCGA data, however, the results are still preliminary, further *in vivo* and *in vitro* experiments are needed to validate the role of sirtuins in KIRC. Despite these limitations, this study might be helpful to guide further investigation of sirtuins in KIRC.

In conclusion, we systematically analyzed the prognostic value and expression of sirtuins in multiple cancer types and provided a thorough understanding of the heterogeneity and complexity of the roles of sirtuins in cancer. Our results indicated that SIRT1, SIRT3, SIRT4, and SIRT5 may play a beneficial role in KIRC, while SIRT6 and SIRT7 may act as unfavorable factors in KIRC. Further studies are required to validate our findings and promote the clinical utility of sirtuins serving as prognostic indicators or therapeutic targets for cancer.

## Data Availability Statement

Publicly available datasets were analyzed in this study. This data can be found here: TCGA (https://portal.gdc.cancer.gov/).

## Author Contributions

YT and NL developed the idea and designed the research. YT analyzed the data. YT and FP drafted the manuscript. BL and GG revised the writing. All authors read and approved the submitted version.

### Conflict of Interest

The authors declare that the research was conducted in the absence of any commercial or financial relationships that could be construed as a potential conflict of interest.
