# Supplementary material for: Integrative Analysis of Sirtuins and Their Prognostic Significance in Clear Cell Renal Cell Carcinoma
Source: Front Oncol. 2020 Feb 25;10:218. doi: 10.3389/fonc.2020.00218 (PMC7052292; doi:10.3389/fonc.2020.00218)
Supplement: Supplementary file 1 [file Table_1.DOCX]

*Supplementary material*

**Supplementary Table1:** Pan-cancer survival analysis of sirtuins
